# Whole-Blood Transcriptome Unveils Altered Immune Response in Acute Myocardial Infarction Patients With Aortic Valve Sclerosis

**DOI:** 10.1161/ATVBAHA.123.320106

**Published:** 2023-12-21

**Authors:** Luca Piacentini, Veronika A. Myasoedova, Mattia Chiesa, Chiara Vavassori, Donato Moschetta, Vincenza Valerio, Gloria Giovanetti, Ilaria Massaiu, Nicola Cosentino, Giancarlo Marenzi, Paolo Poggio, Gualtiero I. Colombo

**Affiliations:** Centro Cardiologico Monzino, IRCCS, Milan Italy (L.P., V.A.M., M.C., C.V., D.M., V.V., G.G., I.M., N.C., G.M., P.P., G.I.C.).; Department of Electronics, Information and Biomedical Engineering, Politecnico di Milano, Milan, Italy (M.C.).

**Keywords:** acute myocardial infarction, aortic valve sclerosis, cardiovascular adverse events, immune response, inflammation

## Abstract

**BACKGROUND:**

Aortic valve sclerosis (AVSc) presents similar pathogenetic mechanisms to coronary artery disease and is associated with short- and long-term mortality in patients with coronary artery disease. Evidence of AVSc-specific pathophysiological traits in acute myocardial infarction (AMI) is currently lacking. Thus, we aimed to identify a blood-based transcriptional signature that could differentiate AVSc from no-AVSc patients during AMI.

**METHODS:**

Whole-blood transcriptome of AVSc (n=44) and no-AVSc (n=66) patients with AMI was assessed by RNA sequencing on hospital admission. Feature selection, differential expression, and enrichment analyses were performed to identify gene expression patterns discriminating AVSc from no-AVSc and infer functional associations. Multivariable Cox regression analysis was used to estimate the hazard ratios of cardiovascular events in AVSc versus no-AVSc patients.

**RESULTS:**

This cross-sectional study identified a panel of 100 informative genes capable of distinguishing AVSc from no-AVSc patients with 94% accuracy. Further analysis revealed significant mean differences in 143 genes, of which 30 genes withstood correction for age and previous AMI or coronary interventions. Functional inference unveiled a significant association between AVSc and key biological processes, including acute inflammatory responses, type I IFN (interferon) response, platelet activation, and hemostasis. Notably, patients with AMI with AVSc exhibited a significantly higher incidence of adverse cardiovascular events during a 10-year follow-up period, with a full adjusted hazard ratio of 2.4 (95% CI, 1.3–4.5).

**CONCLUSIONS:**

Our findings shed light on the molecular mechanisms underlying AVSc and provide potential prognostic insights for patients with AMI with AVSc. During AMI, patients with AVSc showed increased type I IFN (interferon) response and earlier adverse cardiovascular outcomes. Novel pharmacological therapies aiming at limiting type I IFN response during or immediately after AMI might improve poor cardiovascular outcomes of patients with AMI with AVSc.

**Highlights**

- Our genome-wide molecular profiling revealed that blood transcriptome of patients with acute myocardial infarction (AMI) is considerably different between those with aortic valve sclerosis (AVSc) and no-AVSc.
- Increased type I IFN (interferon) response, in patients with AMI with AVSc, is altered and associated with adverse cardiovascular outcomes, providing reliable information for better risk stratification and management of this subclass of patients with AMI.
- Pharmacological therapies focused in inhibiting the type I IFN response during or immediately after AMI might be proposed as a new therapeutic target for improving the medium- and long-term clinical outcomes in patients with AMI with AVSc.

Previous observations have shown that aortic valve sclerosis (AVSc), which is characterized by focal aortic valve thickening,^1^ presents similar pathogenetic mechanisms to coronary artery disease (CAD), such as endothelial dysfunction, chronic inflammatory infiltrates, deposition of lipoproteins, increased oxidative stress, and calcification.^2^ Many studies have shown that independent risk factors related to CAD (ie, age, hypertension, dyslipidemia, and diabetes) are also associated with the development and progression of AVSc.^2^

Consistently, patients with AVSc have been frequently reported to concurrently have underlying CAD, either subclinical or overt, and AVSc has been associated with both short- and long-term mortality in patients with CAD.^3,4^ These common features between AVSc and CAD have prompted clinicians to evaluate the effectiveness of more aggressive pharmacological treatments to tackle both conditions at once.^4^ Indeed, the detection of AVSc could help physicians to make timely decisions to prevent further fatal and nonfatal ischemic events (ie, acute myocardial infarction [AMI]). However, evidence of pathophysiological traits that specifically characterize AVSc in the context of an ongoing AMI is currently lacking.

Nowadays, the availability of rapid and high-throughput sequencing platforms has facilitated molecular phenotyping of disease states (eg, through circulating transcriptome analysis). Global analysis of gene expression such as RNA sequencing represents a paradigm shift from the traditional single-molecule approach for the evaluation of gene regulatory networks, facilitating the recognition of dysregulated genes, and the identification of genes that may be disease biomarkers or therapeutic targets.^5^ We have previously demonstrated the effectiveness of circulating transcriptome analysis in identifying signatures capable of discriminating clinical phenotypes in the context of AMI.^6^

Therefore, since patients with AMI with AVSc may have a worse clinical presentation than patients with no-AVSc patients,^7^ we tested whether whole-blood transcriptome profiling could discriminate between the 2 subgroups, and provide insight into the underlying pathophysiological landscape that differentiates AVSc from no-AVSc patients. We performed RNA sequencing on >100 patients hospitalized for AMI to obtain a genome-wide profiling that could unveil the underlying pathophysiological landscape affecting patients with AVSc.

## METHODS

### Data Availability

Anonymized RNA sequencing data have been made publicly available in the National Center for Biotechnology Information Gene Expression Omnibus repository and can be accessed at https://www.ncbi.nlm.nih.gov/geo/query/acc.cgi?acc=GSE218474.

Anonymized metadata and raw data are available in the Zenodo repository and can accessed through a direct request to the corresponding author (https://doi.org/10.5281/zenodo.10201186).

R codes used for the analyses have been made publicly available in GitHub repository and can be accessed at https://github.com/BioinfoMonzino/Piacentini.et.al.ATVB.

### Study Population

This is a cross-sectional observational study (cf. STROBE checklist in the Supplemental Material). We enrolled 120 patients admitted with ST-segment elevation myocardial infarction (STEMI) or non-ST-segment elevation myocardial infarction (NSTEMI) at the Centro Cardiologico Monzino IRCCS in Milan, Italy, between 2012 and 2015.^6^ Of these, 110 patients, for whom a valid ECG was available to assess the presence or absence of AVSc, were included in this study. STEMI and NSTEMI were defined according to the third universal definition of myocardial infarction.^8^ In particular, STEMI and NSTEMI identify patients with AMI but with different ECG changes at presentation that reflect total or subtotal coronary artery occlusion, respectively. Exclusion criteria included AMI with hemodynamic (Killip class IV), electrical (sustained ventricular tachycardia/ventricular fibrillation/atrial fibrillation with rapid ventricular response), and mechanical (rupture of the left ventricular free wall, rupture of the interventricular septum, and acute mitral regurgitation due to papillary muscle necrosis) complications at hospital presentation; chronic atrial fibrillation; systemic diseases, such as malignancy, infections, or autoimmune diseases. Patients with STEMI and NSTEMI with AVSc were referred to as the AVSc group, whereas those without AVSc were included in the no-AVSc group.

Information on cardiovascular events (ie, rehospitalization for acute heart failure or AMI, unplanned percutaneous or surgical coronary revascularization, cerebrovascular events, and cardiovascular mortality) was retrieved from medical records of hospitalizations and outpatient visits over a 10-year follow-up.

The study protocol followed the principles of the Declaration of Helsinki. The Ethics Committee of the IRCCS Centro Cardiologico Monzino approved the study protocol (Centro Cardiologico Monzino-PA-19062012). All enrolled patients signed written informed consent. Participants also consented to share their deidentified information.

### Echocardiographic Evaluation

Experienced cardiologists of Centro Cardiologico Monzino performed echocardiographic scans according to current guidelines.^9–11^ As previously performed by us,^4^ the morphology and function of the aortic valve were assessed from the recorded echocardiographic images to evaluate the presence of AVSc, expressed as a dichotomous variable (yes or no). In cases where echocardiographic analysis of the aortic valve was not detailed on admission, we evaluated echocardiographic images before discharge of the patients. AVSc was identified according to criteria described by Gharacholou et al,^1^ that is, irregular, nonuniform thickening of portions of the aortic valve leaflets or commissures, or both; with or without thickened portions of the aortic valve with an appearance suggesting calcification (ie, bright echoes); nonrestricted or minimally restricted opening of the aortic cusps; and peak continuous wave Doppler velocity across the valve <2 m/s.

### Blood Sample Collection and RNA Isolation

Peripheral blood samples drawn from an antecubital vein were collected into Tempus Blood RNA tubes containing RNA stabilizing reagents (Thermo Fisher Scientific, catalog no.: 342792) at the time of hospital admission, before any medical intervention.

Total RNA was isolated using the Tempus Spin RNA Isolation Kit, following the manufacturer’s instructions (Thermo Fisher Scientific, catalog no.: 4380204). Isolated RNA was treated with DNAse (TURBO DNAse; Thermo Fisher Scientific, catalog no.: AM2238) to remove genomic DNA contamination.

### RNA Sequencing

Whole-blood gene expression profiles were assessed using globin-depleted, poly(A)+ enriched RNA. Libraries were prepared and pooled together by a multiplex library RNA barcoding system and sequenced using the Sequencing by Oligonucleotide Ligation and Detection approach (Applied Biosystems). Templates were paired-end sequenced (75 forward and 35 reverse base pairs). Raw reads were mapped against the GRCh38 Human Genome reference (release 99) with the Spliced Transcripts Alignment to a Reference v2.7.5c software^12^ and with Bowtie2 v2.4.1,^13^ whereas gene expression quantification was computed by featureCounts v2.0.1.^14^

Raw counts were then used for data exploration by using the EDASeq and DaMiRseq R/Bioconductor packages.^15,16^

### Feature Selection

Selection of informative variables for discriminating patients with or without AVSc was performed with a genetic algorithm specifically developed to work with high-dimensional data, implemented as the GARS (Genetic Algorithm for the Identification of Robust Subsets) R/Bioconductor package.^17^ The optimal number of informative genes was chosen by running several tests, varying the length of the feature set (ie, chromosome length), ranging from a minimum of 10 to a maximum of 200 genes. The feature set with the highest fit score was then tested to assess AVSc versus no-AVSc discrimination performance through the DaMiR.EnsembleLearning function of the DaMiRseq R/Bioconductor package.^16^

### Differential Gene Expression Analysis

Differential expression analysis (DEA) was performed on filtered raw counts data (see Supplemental Material for details on filtering criteria) using negative binomial generalized linear models (DESeq2 R/Bioconductor package).^18^ The presence of unknown confounding factors (aka latent variables) to properly adjust the statistical models for DEA was estimated using the EDASeq and RUVSeq R/Bioconductor packages.^15,19^ Details on the identification of latent variables are reported in the data adjustment section of the Supplemental Material. Different models were thus designed and tested to find specific gene expression differences between AVSc versus no-AVSc patients. The zero models (ModØ) included the abovementioned latent variables (n=13), the AMI type (ie, STEMI and NSTEMI), and the AVSc factor (ie, the primary variable of interest). The other models included, in addition to the ModØ variables, (1) the covariate age (Mod1) and (2) age plus the previous AMI or percutaneous coronary intervention (PCI) and coronary artery bypass graft (CABG) interventions (yes/no) factor variable (Mod2).

Gene expression was deemed as significantly different between AVSc and no-AVSc at a false discovery rate–adjusted *P*<0.05 and |log2-fold changes| >0.5. The Benjamini-Hochberg method was used to control the false discovery rate. The EnhancedVolcano R/Bioconductor package was used to graphically summaries the DEA results by drawing scatter plots of fold change differences versus significance (volcano plots).

The robustness of the DEA results was assessed by exploring the histograms of the *P* value distribution. For clustering analysis, a scatterplot of 2 coordinates (X1 and X2) obtained from the multidimensional scaling performed on the whole gene expression matrix of the log2-transformed normalized counts was drawn.

### Power Analysis

Using the rnapower function of the RNASeqPower R/Bioconductor package, we estimated that our study had a statistical power of 91% with a sample size of 110 patients. The parameters set for this calculation were as follows: depth=10; n=66 (sample size of the controls), n2=44 (sample size of the cases), coefficient of variation=0.2 (estimated from sequencing data obtained in previous studies^6^), effect size=1.4 (on the linear scale, corresponding to |log_2_ fold change=0.5), and alpha=10^−^^3^.

### Functional Enrichment Analysis on Genome-Wide Expression Profiles

To associate specific biological functions with AVSc and no-AVSc phenotypes, we benefited from prior biological knowledge on genes grouped by gene ontology (GO) biological processes (BP) to perform gene set enrichment analysis (software v4.1.0).^20^ To visually interpret biological data, networks of the most significant GO-BP (at a false discovery rate <0.05) were drawn through the Enrichment Map software v3.3.2,^21^ implemented as an app in the Cytoscape v3.8.2 platform.^22^

Similarly, a cell-type enrichment analysis to associate specific immune cell types with AVSc and no-AVSc phenotypes was performed based on a gene set collection of 22 subsets of human hematopoietic cell types.^23^

### Statistical Analysis

All the statistical analyses and graphs were performed by the R software v4.1.1. Missing data for a few clinical variables (Table S1) have been imputed using the iterative robust model-based imputation (irmi function) method of the VIM R package. Demographic and clinical categorical data are presented as counts and percent, continuous data as medians and interquartile range (Q1–Q3). Categorical and continuous variables were compared by χ^2^ and Wilcoxon rank sum (2-sided) test, respectively. Assessment for the presence of correlations among demographic and clinical variables was performed by computing the Spearman correlation (rho). A *P*<0.05 was considered statistically significant.

Kaplan-Meier analysis was used to generate time-to-event curves for cardiovascular events in patients with and without AVSc and the log-rank test was used to compare the strata. Multivariable Cox regression analysis was implemented to consider possible confounding effects. Cox proportional hazards assumptions were tested using Schoenfeld residuals, while deviance and Martingale residuals were used to generate diagnostic plots to examine the possible presence of outliers and nonlinearity of continuous variables, respectively.

To test expression differences of selected genes between patients with and without AVSc suffering from early or late (<5 and >5 years, respectively) cardiovascular events after AMI, a linear model for the estimation of marginal means for factor combinations was implemented. False discovery rate multiplicity adjustment for tests was applied.

Details on RNA processing protocol, library preparation, RNA sequencing, and data processing and analysis parameters are available in the Supplemental Material.

## RESULTS

### Study Population Characterization

Table 1 reports baseline demographic, clinical, and laboratory features of the 44 AVSc and 66 no-AVSc patients studied. The proportions of men to women in both AVSc and no-AVSc patients reflect published epidemiological data (ie, ≈3:1 in both groups), and AVSc subjects are 14 years older than no-AVSc ones in our cohort, in accordance with published literature.^7^ We also observed a significantly higher fraction of patients with previous AMI, PCI, or CABG, that is, 3× more, compared with no-AVSc. Patients with AVSc also had significantly lower leukocyte values than no-AVSc patients (particularly lymphocytes), as well as total cholesterol, triglycerides, and estimated glomerular filtration rate levels. The differences observed between the 2 groups for the latter variables can be explained, at least in part, by their significant correlation (0.27<|rho<0.43; *P*<0.01) with age, which remains the variable with the highest difference between AVSc and no-AVSc patients (Figure S1). Notably, only age and previous AMI/PCI/CABG withstand adjustment for multiple testing using the Bonferroni method (adj. *P*<0.01). In contrast, we found no significant imbalance between AVSc and no-AVSc in the proportion of patients with STEMI or NSTEMI (consistently with on admission and peak cardiac troponin I values), drug therapies on admission, time-to-presentation after onset of AMI symptoms, and left ventricular ejection fraction.

**Table 1.**
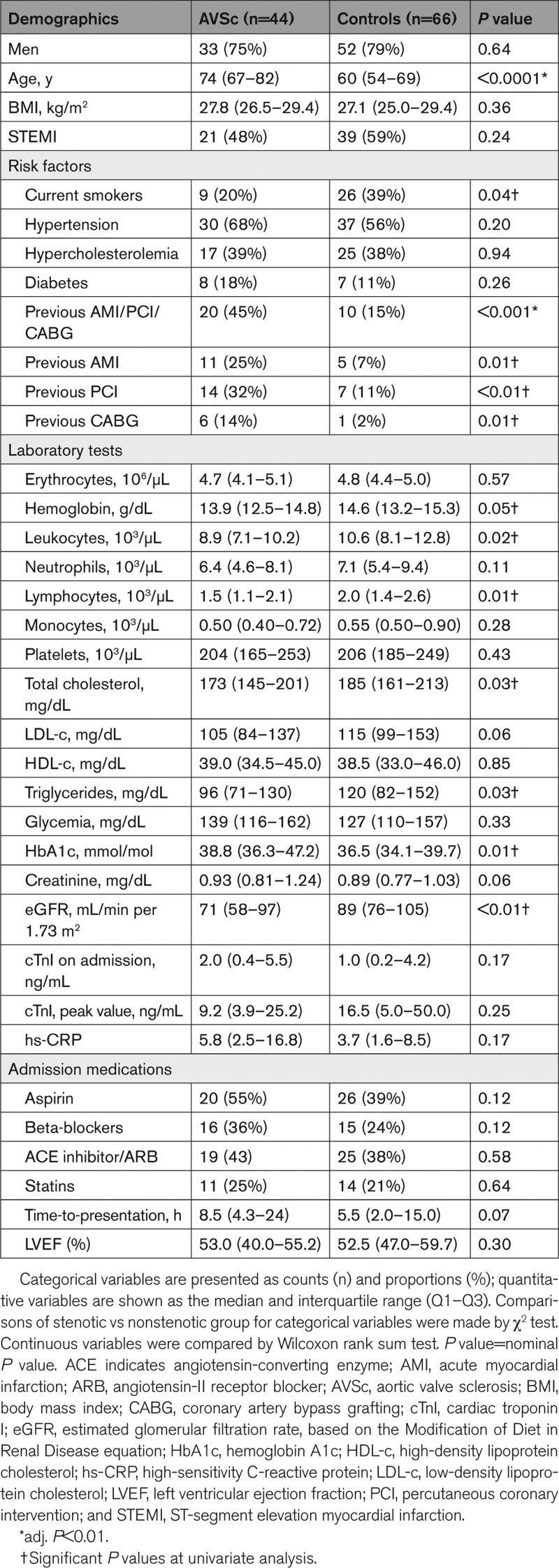
Patients’ Characteristics

### Gene Expression Dataset

**Figure 1.**
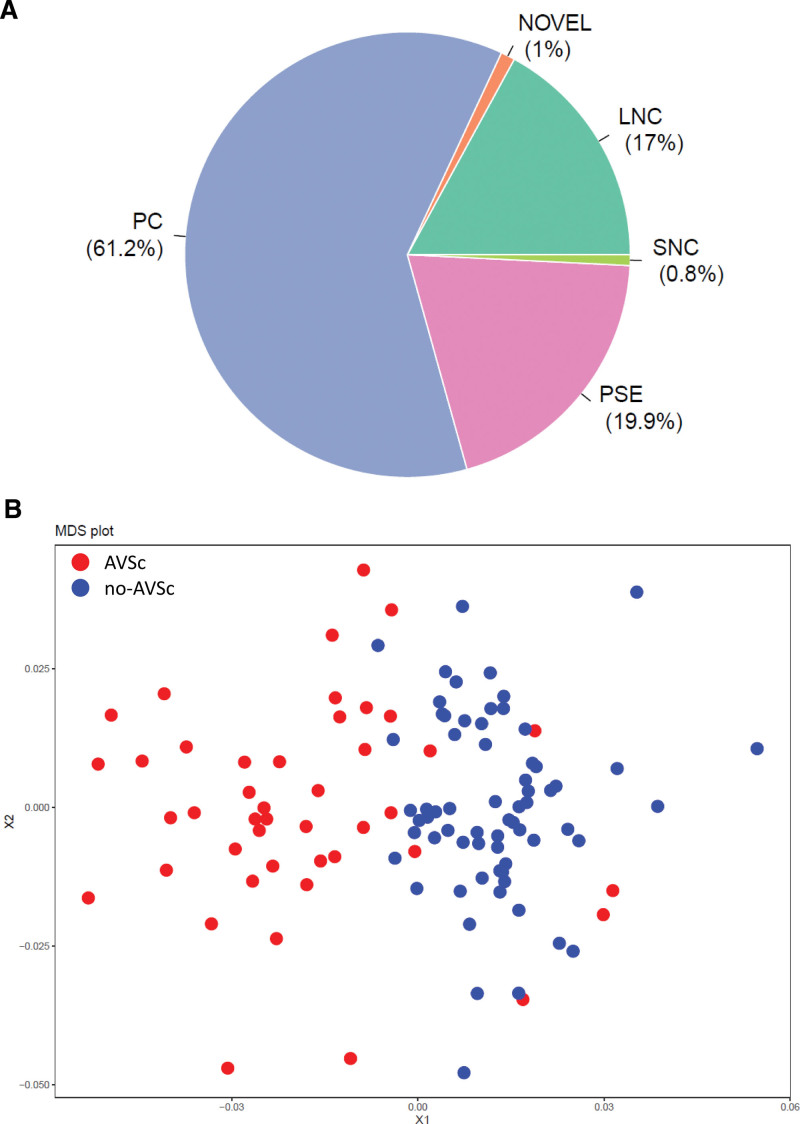
**Gene expression dataset and feature selection. A**, Pie chart shows the relative abundance of the different RNA biotypes of the entire set of expressed genes. **B**, Scatterplot of the 2 coordinates (X1 and X2) obtained from the multidimensional scaling performed on the 100 genes identified by applying feature selection based on genetic algorithm (ie, GARS [Genetic Algorithm for the Identification of Robust Subsets] R/Bioconductor package). Colors refer to aortic valve sclerosis (AVSc) and no-AVSc patients (red and blue, respectively). LNC indicates long noncoding; NOVEL, putative novel genes; PC, protein coding; PSE, pseudogenes; and SNC, small noncoding.

A mean of 36.2±7.5 million reads per sample mapped against the human genome reference, including those located at unannotated loci. We identified 25 509 expressed genes (filtering criteria in the Methods section of the Supplemental Material), of which 15 623 were protein-coding genes, 5079 pseudogenes, 4346 long noncoding, 198 short noncoding, and 263 putative novel genes (Figure 1A). By using the entire gene expression matrix, adjusted for latent variables, we got an overview of the expression data and inspected possible subsample grouping. The scatterplot of the 2 multidimensional scaling coordinates, which aims to visualize the level of similarity of individual observations of a dataset in a 2-dimensional space, showed a rather homogeneous sample distribution with only a very slight trend towards the distinction of AVSc from no-AVSc patients (Figure S2).

### AVSc and no-AVSc Patient Discrimination Through Feature Selection on Whole-Blood Transcriptome

To test the possibility of unambiguously distinguishing AVSc from no-AVSc patients by using peripheral blood transcriptome data, we applied a feature selection method to identify a set of informative genes, by excluding redundant or ineffective variables. We obtained a set of 100 genes by which the resulting multidimensional scaling plot showed a much sharper distinction between AVSc and no-AVSc patients than that one obtained by using the entire dataset (Figure 1B; Supplemental Material: (a) Feature Selection). By applying a machine learning method (Methods section in the Supplemental Material), we observed that this gene set was able to discriminate AVSc from no-AVSc patients with 94% accuracy, suggesting that blood gene expression patterns are associated with AVSc in patients with AMI.

This method, while effective in distinguishing patient phenotype (AVSc and no-AVSc) at blood transcriptome level, is not suitable for identifying differentially expressed (DE) and functionally related genes. Hence, to characterize the underlying pathophysiological mechanism(s) of the 2 groups of patients, we performed DEA followed by gene set enrichment analysis on the entire list of the DEA results.

### Whole-Blood DEA Between AVSc and no-AVSc Patients

**Figure 2.**
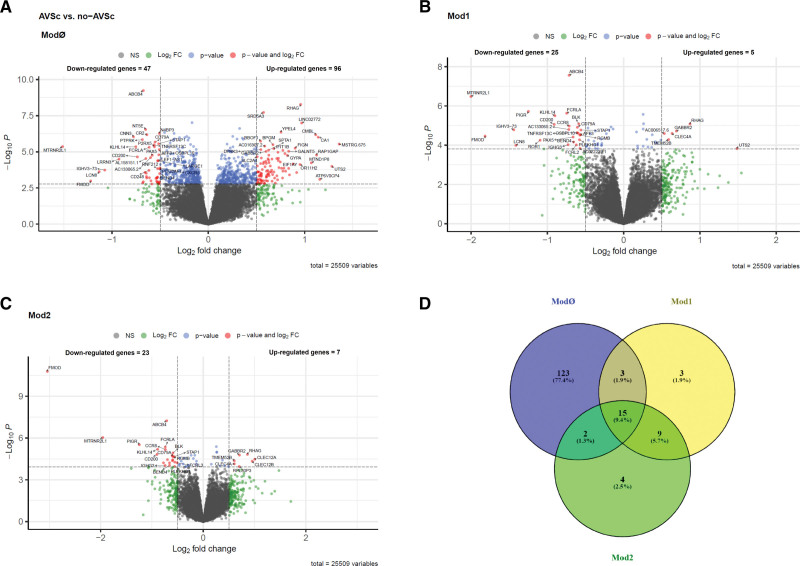
**Volcano plots and Venn diagram.** Scatterplot of log2fold change (FC) vs the significance (*x* and *y* axis, respectively) for the comparison between aortic valve sclerosis (AVSc) and no-AVSc patients found from model (Mod)Ø (**A**), Mod1 (**B**), and Mod2 (**C**). Plot colors refers to differentially expressed (DE) genes (red), genes with adj. *P*<0.05 but |log2FC| <0.5 (blue), genes with |log2FC|>0.5 but adj. *P*>0.05 (green); nonsignificant DE genes (gray). Vertical and horizontal dashed lines represent log2FC and adj. *P* value thresholds, respectively. **D**, The intersection of DE genes (adj. *P*<0.05 and |log2FC| >0.5) obtained from the different statistical models. NS indicates not significant.

DEA, adjusted for the effects of confounders (ie, latent variables) and relevant clinical variables, showed specific mean changes in whole-blood gene expression between AVSc and no-AVSc patients. We included age and previous AMI or PCI/CABG interventions for model adjustment since these were the clinical variables with the most marked difference between the AVSc and no-AVSc groups (Table 1) and could have affected the association between gene expression and AVSc phenotype in patients with AMI. We reported the overall results and statistics for each statistical model in the Supplemental Material: (b) differential expression analysis, and summarized the main findings in Table 2. Briefly, we found a substantial number of DE genes (n=143, of which 96 overexpressed and 47 underexpressed; adj. *P*<0.05 and |log_2_ fold change|>0.5) by comparing AVSc versus no-AVSc (ModØ; Figure 2A). Importantly, 30 DE genes were shown to be associated with AVSc regardless of the effect of age (Mod1), as they remained significant even after model adjustment (Figure 2B). Another 30 genes (partially overlapping with those identified in Mod1) were DE independently of the concurrent effects of age and previous AMI or PCI/CABG interventions (Mod2; Figure 2C). Intersection among genes found DE by the 3 models is shown in Figure 2D. The *P* values for the 3 DEA models showed a uniformly flat distribution over the entire unit interval (null *P* values) with a peak near zero (*P* values for alternative hypotheses), which fitted with that expected for the truly DE genes (Figure S3A through S3C).

**Table 2.**
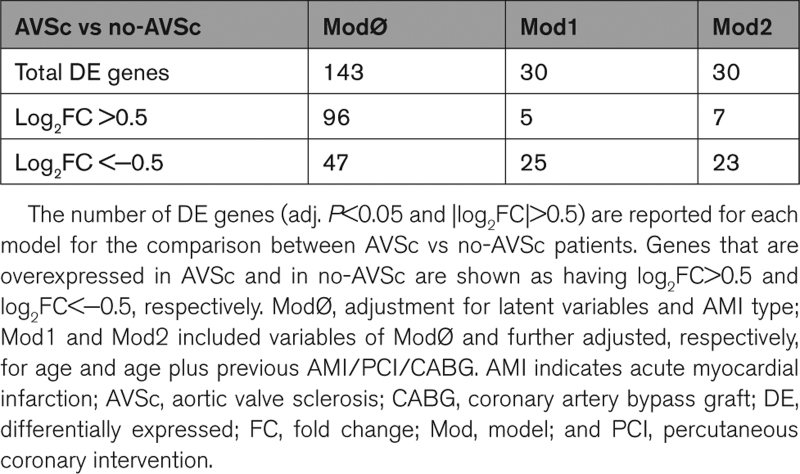
Summary of Differential Expression Analysis

### Functional Inferences From Genome-Wide DEA

**Figure 3.**
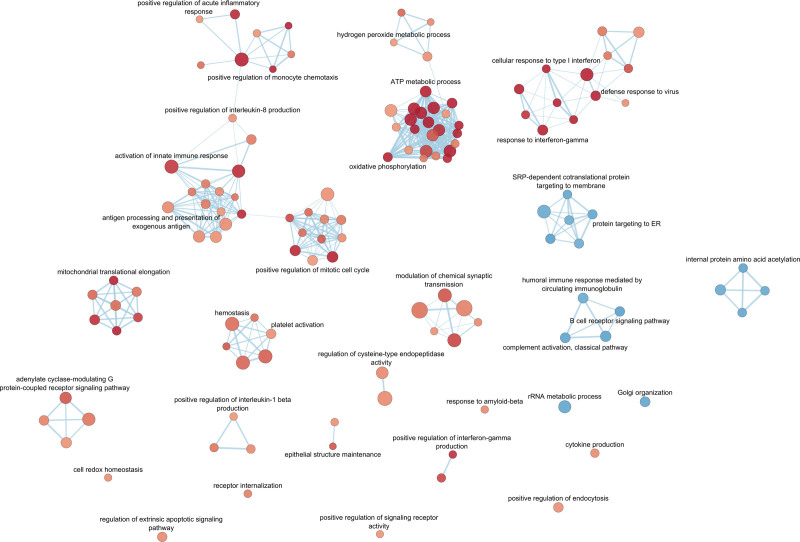
**Functional enrichment network.** The enrichment network shows the gene ontology (GO)-biological processes (BP) gene sets (nodes) that are significantly associated (false discovery rate <0.05) either with aortic valve sclerosis (AVSc) and no-AVSc patients. The node color refers to the association with the phenotype (AVSc, red and no-AVSc, blue); node gradient color is proportional to the gene set normalized enrichment score (NES), from lower (light) to higher (dark); node size is proportional to the gene set size. Labels of overview gene set terms grouping nodes with similar meaning are shown. Edges connect related GO-BPs. Edge thickness is proportional to the similarity between 2 GO-BPs, for a cutoff=0.25 of the combined Jaccard plus Overlap coefficient. ER indicates endoplasmic reticulum; and SRP, signal recognition particle.

To infer biological functions characterizing the patients with AVSc, we performed a gene set enrichment analysis on the DEA result obtained from the fully adjusted model (Mod2). We found a considerable number of significant GO-BPs (n=129) that were over-represented or under-represented in the AVSc group (Supplemental Material: (c) GSEA). The most representative GO-BPs that were positively associated with the AVSc phenotype were suggestive of altered innate immune response and inflammation, as the ones related to both type I and gamma IFN (interferon) response, positive regulation of mononuclear cell migration, acute inflammatory response, interleukin-8 and -1 beta production, antigen processing and presentation, cell cycle, platelet activation, and hemostasis. Conversely, the most representative GO-BPs negatively associated with AVSc were mainly related to humoral immune response, including immunoglobulin-mediated immune response, B cell receptor signaling pathway, complement activation (classical pathway), and protein acetylation and co-translational protein targeting. To facilitate the overall data interpretation and visualize the relationships among GO-BPs, we summarized the results of gene set enrichment analysis into an enrichment network highlighting gene set terms that recapitulated the functions of closely related GO-BPs (Figure 3).

### Cell-Type Enrichment

**Figure 4.**
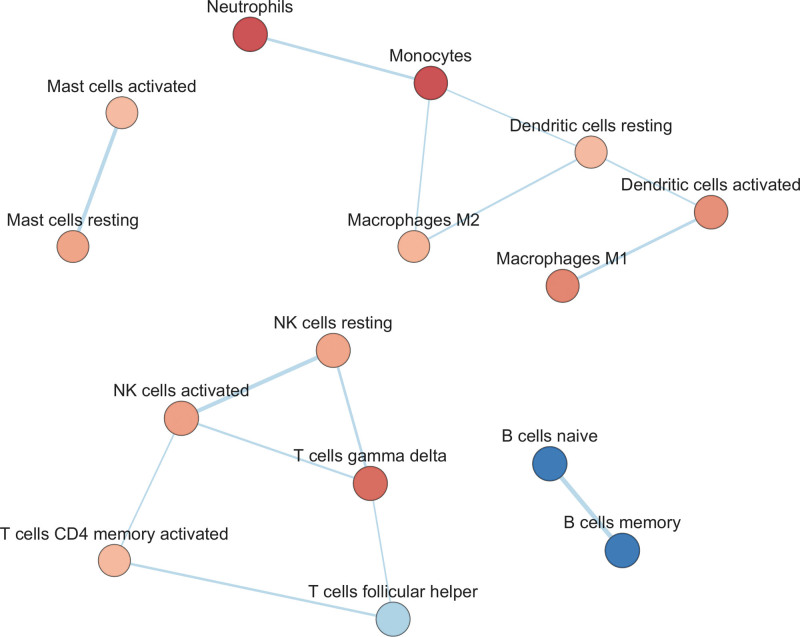
**Cell-type enrichment network.** The network shows the significant gene sets related to immune cell types (false discovery rate <0.05) that are enriched in the comparison between aortic valve sclerosis (AVSc) and no-AVSc patients. The node color refers to the association with the phenotype (AVSc, red and no-AVSc, blue); node gradient color is proportional to the cell-set normalized enrichment score (NES), from lower (light) to higher (dark); node size is proportional to the cell-set size. Edges connect related cell type. Edge thickness is proportional to the similarity between 2 cell-type, for a cutoff=0.25 of the combined Jaccard plus Overlap coefficient. NK indicates natural killer.

Since altered immune responses were inferred in patients with AVSc, we further investigated whether specific immune cell types could be differentially associated with the AVSc phenotype in patients with AMI (functional enrichment analysis in the Methods section). We observed that AVSc was significantly associated with innate immune cells, such as neutrophils, monocytes, mast cells, and dendritic cells, and with well-defined subgroups of lymphoid cells, including resting and activated natural killer cells, CD4+ memory, and gamma/delta T-lymphocytes. AVSc was instead negatively associated with both naive and memory B cells and follicular helper T-cells (Supplemental Material: (c) GSEA; Figure 4). These findings corroborate the abovementioned associations with BPs.

### Adverse Cardiovascular Events in Patients With AMI

We collected information on cardiovascular events for 89 patients with AMI (n=35 AVSc and n=54 no-AVSC patients; 21 were lost at follow-up) during a median follow-up of 5.6 years (interquartile range, 2.4–7.7). We recorded 56 cardiovascular events, 32 in the AVSc group and 24 in the no-AVSc group (91.4% versus 44.5%, respectively; *P*<0.0001; Table S2).

**Figure 5.**
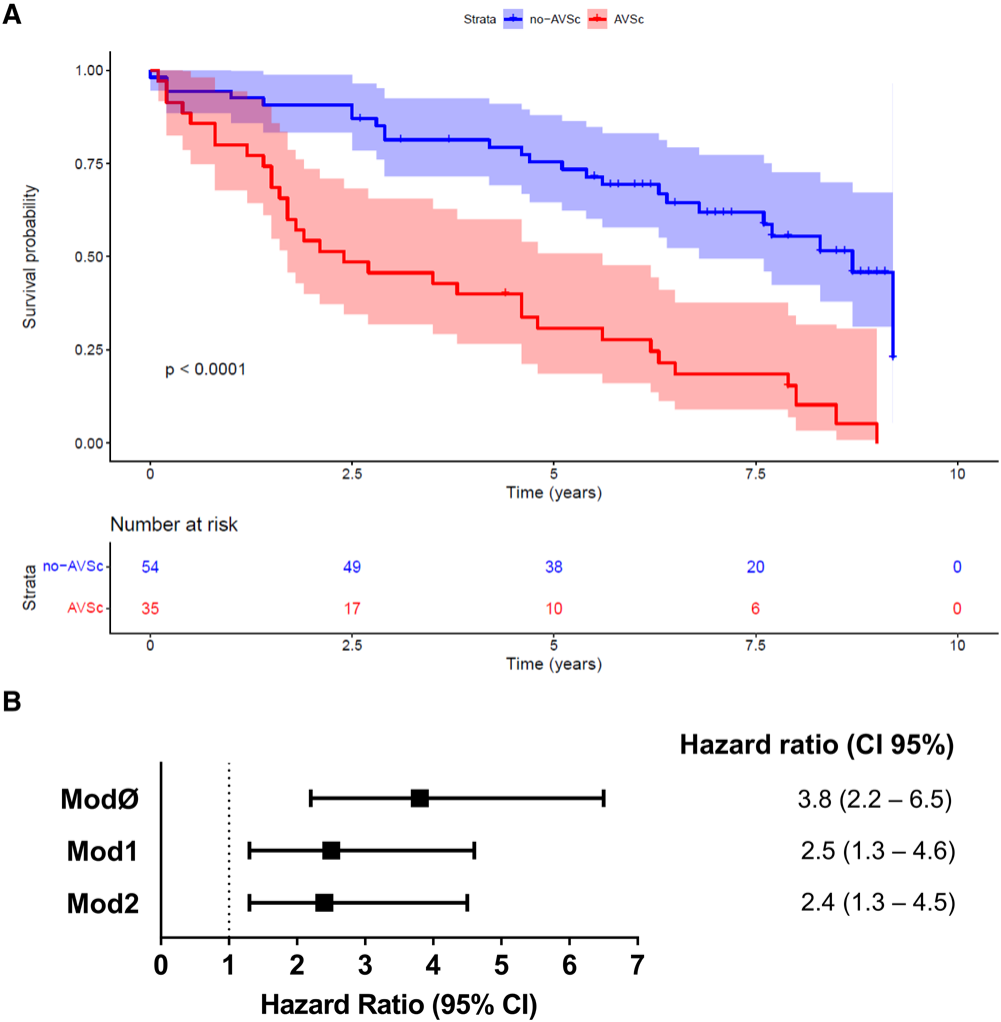
**Adverse cardiovascular events in patients with acute myocardial infarction (AMI). A**, Kaplan-Meier curves show the event’s probability of cardiovascular events in patients with AMI with aortic valve sclerosis (AVSc) vs patients with AMI without AVSc. **B**, Cox regression analyses show the hazard ratios (HRs) adjusted for AMI type only (ModØ), for AMI type and age (Mod1), and for AMI type, age plus previous cardiovascular events (Mod2).

Kaplan-Meier curves showed a significantly increased event rate in patients with AMI with AVSc than in no-AVSc ones (*P*<0.0001; Figure 5A). Hazard ratios associated with AVSc are shown in Figure 5B. Regression models were adjusted for the same clinical variables used for DEA. The unadjusted Cox regression analysis (ModØ) revealed that patients with AVSc had an increased risk of cardiovascular events at long-term follow-up (hazard ratio, 3.8 [95% CI, 2.2–6.5]; *P*<0.0001). This association withstood all the adjustments (ie, age and previous AMI/PCI/CABG; Mod2: HR, 2.4 [95% CI, 1.3–4.5]; *P*=0.007). Table S3 shows the complete Cox regression results for the fully adjusted model (Mod2). Assumptions for Cox proportional model have been tested and summarized in the Supplemental Material (Table S4; Figures S4 and S5).

**Figure 6.**
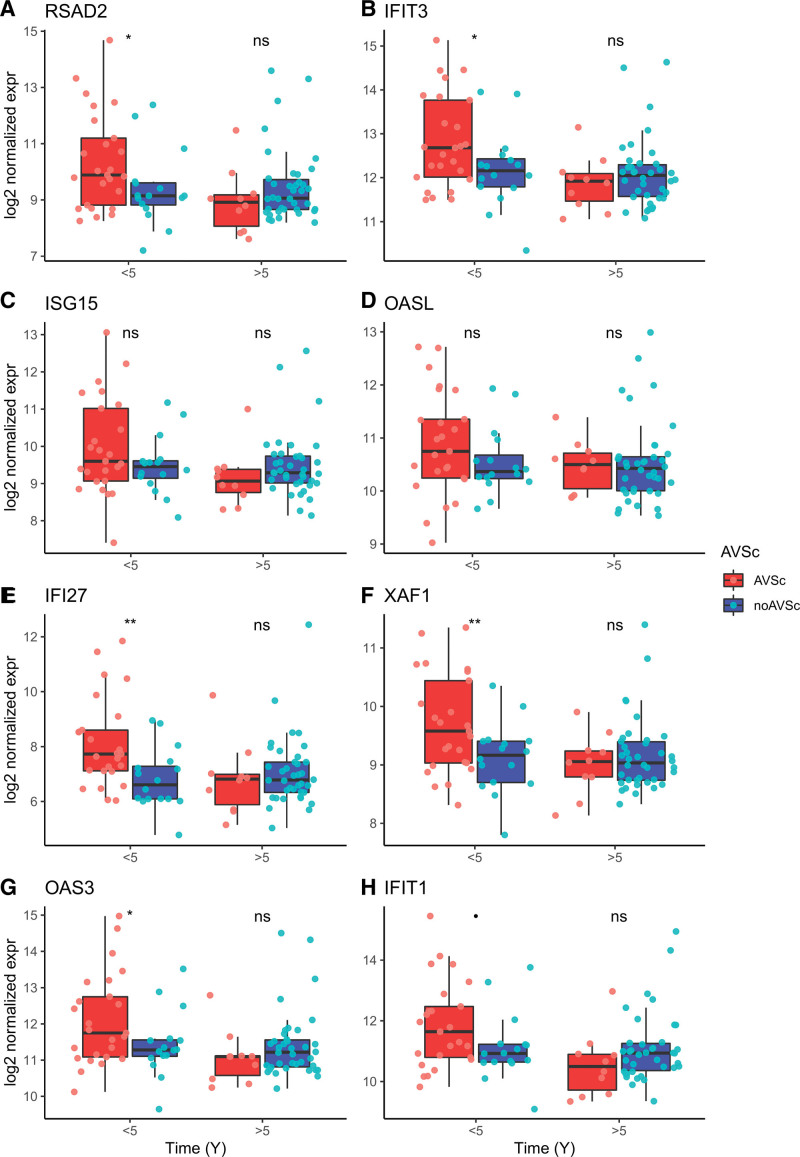
**Box plots of relevant type I IFN (interferon) response genes.** Box plots show the differences over the time-to-cardiovascular event (<5 and >5 years; *x* axis) of the normalized expression values (*y* axis) of the type I IFN response genes in aortic valve sclerosis (AVSc) and no-AVSc patients assessed on blood sample collected at hospital presentation. The 8 genes shown (**A–H**) were selected as they presented the highest combined-ranked score (cs, that is, log2-fold-change [FC]×−log10[*P* value]) among the type I IFN response genes as resulted by the differential expression analysis of AVSc vs no-AVSC in the full adjusted statistical model (Mod2). Number of patients for time-to-cardiovascular event comparisons were for <5 years: AVSc=25, no-AVSc=16; and for >5 years, AVSc=10, no-AVSc=38. Box (red and blue) and dots (pink and light blue) colors refer to AVSc and no-AVSc patients, respectively. Stars mark significant differences for post hoc tests with *P* values: **<0.01; *<0.05; •<0.1; ns indicates nonsignificant difference. IFI27 indicates interferon alpha-inducible protein 27; IFIT1, interferon-induced protein with tetratricopeptide repeats 1; IFIT3, interferon-induced protein with tetratricopeptide repeats 3; ISG15, ISG15 ubiquitin-like modifier; OAS3, 2’-5’-oligoadenylate synthetase 3; OASL, 2’-5’-oligoadenylate synthetase-like; RSAD2, radical S-adenosyl methionine domain containing 2; and XAF1, XIAP-associated factor 1.

Notably, we found that the most relevant DE genes associated with type I IFN response, such as RSAD2 (radical S-adenosyl methionine domain containing 2), IFIT3 (interferon-induced protein with tetratricopeptide repeats 3), IFI27 (interferon alpha-inducible protein 27), XAF1 (XIAP-associated factor 1), and OAS3 (2’-5’-oligoadenylate synthetase 3), showed significantly higher average expression levels in the subgroup of patients with AVSc who had accelerated adverse clinical outcomes (ie, major adverse cardiovascular events within 5 years) compared with those who had a later event or no-AVSc patients (Figure 6). This finding suggested a direct relationship between the pattern of response to type I IFNs and the outcome of patients with AVSc after AMI.

We observed similar results for DE genes related to the positive regulation of IL1β (interleukin-1beta), such as NOD2 (nucleotide-binding oligomerization domain containing 2), CASP1 (caspase 1), CLEC7A (C-type lectin domain containing 7A), and FZD5 (frizzled class receptor 5), which were overexpressed in patients with AVSc who had cardiovascular events within 5 years but not in those who had later events (Figure S6). CARD16 (caspase recruitment domain family member 16) and CD33 (sialic acid-binding Ig-like lectin 3) were overexpressed in patients with AVSc with events recorded both within and after 5 years. In contrast, we did not find a clear association of DE genes related to platelet activation and aggregation; we observed a rather different trend. Indeed, although platelet-related processes were robustly associated with the overall AVSc phenotype (Figure 3; Supplemental Material 2C), we did not find an association with accelerated adverse clinical outcomes in patients with AVSc, but 2 genes (CLEC1B [C-type lectin domain family 1 member B] and PDGFRA [platelet-derived growth factor receptor alpha]) were found to be overexpressed in both early and late event patients (Figure S7).

## DISCUSSION

AVSc is thought to share pathogenic mechanisms with CAD, although current research suggests that patients with AVSc have a poorer clinical prognosis than no-AVSc patients, implying that AVSc may have unique pathogenic processes. Here, we tested whether whole-blood gene expression profiling could discriminate between AVSc and no-AVSc patients during AMI and provide insights into functional associations. To our knowledge, this is the first study showing that the blood transcriptome of patients with AMI is considerably different between those with and without AVSc.

First, we found that a specific set of informative genes could distinguish patients with AMI and without AVSc. Second, we found that a substantial fraction (about 20%) of DE genes between AVSc and no-AVSc patients were independent of age, previous AMI, and interventions (ie, PCI or CABG). This suggests that age may account for a relevant part of the AVSc phenotype in our AMI cohort but also that it does not explain all of its complexity. Importantly, the observed differences in overall gene expression showed consistent associations with BPs that could have important pathophysiological relevance (eg, affecting the short- or long-term outcome of these patients). For AVSc, altered BPs mainly fall within the acute immune-inflammatory response and coagulation. In particular, the association of pathways related to increased platelet activation, aggregation, and blood coagulation with AVSc and worse outcomes would suggest that a (marked) prothrombotic state may be responsible, at least in part, for an increased risk of adverse events.^24^ In line with our results, t-PA (tissue-type plasminogen activator), an important player in the homeostasis of coagulation and fibrinolysis, was shown to contribute to AVSc development via cytokine release, leading to increased inflammation and endothelial injury.^25^ Observation from our study may have a clinical perspective since aggressive antithrombotic preventive strategies could be implemented to decrease the recurrence of cardiovascular adverse events after AMI in patients with AVSc.

Another relevant and even more appealing observation is the association between AVSc and cellular response to type I IFNs and activation of the innate immune response. During myocardial infarction, damage-associated molecular patterns are released from dying cells and may contribute to the (innate) inflammatory response by inducing type I IFNs. When dysregulated, the type I IFN system can adversely guide many (autoimmune and nonautoimmune) inflammatory diseases. Along with our data, other studies demonstrated that the response to type I IFNs begins far from the site of injury, that is, in neutrophil and monocyte progenitors in the bone marrow, and that it is detectable in peripheral blood neutrophils and monocytes following myocardial infarction.^26^ Consistently, we also found that neutrophils and monocytes are the most significantly enriched blood cell populations in AVSc compared with no-AVSc. Also, type I IFNs have also been reported to increase the calcification of aortic valve interstitial cells, suggesting a direct role of type I IFN response in the evolution of aortic valve disease.^27^

These observations seem to support the hypothesis of a possible relationship between the effects produced by type I IFNs and the worsening of patient clinical evolution after myocardial infarction, characterizing patients with AMI with AVSc. A preclinical study in mouse models of myocardial infarction has provided a mechanistic model that supports this hypothesis, demonstrating that mice genetically deficient for type I interferon signaling pathway (or receiving anti-type I IFN-signaling drugs) had decreased inflammatory response, attenuated ventricular dilation, and overall improved cardiac performance than control mice.^28^ Consistently, a subsequent study has shown that stimulation of type I interferon production following myocardial ischemia exacerbates reperfusion injury and that inhibition of type I IFNs reduces the ischemia-induced inflammatory response and postischemic reperfusion injury.^29^ Remarkably, we found that the risk of new events within 10 years from the index AMI was significantly higher in AVSc than no-AVSc patients in our cohort, with an even higher risk (within the first 5 years) for patients with high expression of type I IFN response genes. This observation strengthens the hypothesis of a possible mechanistic link between type I IFN response and AVSc patients’ poor outcomes.

We showed that IL1β response genes are also associated with an increased risk of adverse cardiovascular events within 5 years in patients with AVSc. This finding is in line with the evidence that anti-inflammatory therapy (eg, the IL1β inhibitor canakinumab) is effective in reducing the total number of cardiovascular events in patients with prior AMI, as shown in the CANTOS trial (Canakinumab Antiinflammatory Thrombosis Outcome Study).^30,31^ We think that these observations provide a particularly interesting clinical perspective in terms of therapeutic approaches and prevention of adverse events because of the mechanistic aspects related to type I IFNs in AMI and AVSc: the type I IFN and IL-1 cytokine pathways represent distinct types of innate inflammatory responses that may play complementary roles in determining outcome in at-risk patients with AMI.^32^

Indeed, our previous clinical studies, conducted on a much larger cohort, suggest that AVSc is associated with both all-cause and cardiovascular mortality, and AVSc can be considered as an independent prognostic risk marker of adverse outcomes in patients with AMI.^4,33^ Furthermore, our findings are consistent with recent evidence suggesting that patients with AMI and moderate aortic stenosis (a more advanced stage of aortic valve degeneration) experience worse clinical outcomes at 1-year follow-up compared with those with mild or no aortic stenosis.^34^ This adds further support to our observation that patients with AMI and AVSc are at increased risk of adverse cardiovascular outcomes.

Finally, our study unveiled a molecular expression pattern associated with AVSc in the challenging context of AMI. While recognizing the need for further investigation to determine the generalizability of our findings to other conditions, the presence of a recognizable blood-based molecular signature even during the inflammatory storm in AMI suggests that this may be, at least in part, shared in other contexts. Future dedicated studies are warranted to address this issue. In addition, the diversity within our patient population, encompassing various age groups, sexes, and comorbidities, strengthens the potential relevance of our findings to a broader spectrum of patients with AVSc. Furthermore, our findings are consistent with those of previous research,^3,4,35–37^ which reinforce the notion that AVSc is associated with adverse cardiovascular outcomes. By continuing to unravel the intricate molecular mechanisms underlying this condition, our work may contribute to improved diagnosis and screening, offering potential benefits to patients affected by nonstenotic aortic valve fibro-calcification remodeling.

### Study Limitations

Our study had several potential limitations. This is an associative study supported by in vivo observations; hence, mechanistic implications may only be inferred. However, mouse models of myocardial infarction have provided a mechanistic insight that supports the direct role of type I IFNs in cardiovascular outcomes.^28^ In addition, as with any single-center study, the generalizability of our findings to other patient populations or health care settings may be limited. Different coronary stents and antithrombotic agents were used during the study period. Yet, this corresponds to a real-world scenario where patients are treated with different antiplatelet drugs, anticoagulants, and stents according to clinical setting, operator choice, and drug/device availability. No information was available regarding patients’ adherence to treatment during follow-up.

## CONCLUSIONS

The results of our study show that there are specific whole-blood gene expression differences between patients with AMI with and without AVSc. The most representative BPs associated with the AVSc phenotype are suggestive of altered innate immune and inflammatory responses. In addition, cell-type enrichment analysis indicates that patients with AVSc are significantly and positively associated with innate immunity cells, while negatively associated with both naive and memory B cells and follicular helper T-cells, suggesting a clear link between specific cell types and related biological functions. Amplified response of type I IFNs in patients with AVSc was associated with an increased risk of adverse cardiovascular outcomes. Novel pharmacological therapies focused on inhibiting type I IFN response during AMI might be proposed to improve the poor outcomes experienced by patients with AMI with AVSc.

## ARTICLE INFORMATION

### Acknowledgments

L. Piacentini, G. Marenzi, P. Poggio, and G.I. Colombo made substantial contributions to the design of the study. V.A. Myasoedova, C. Vavassori, D. Moschetta, V. Valerio, G. Giovanetti, and N. Cosentino acquired the data. L. Piacentini, M. Chiesa, and I. Massaiu analyzed the data. L. Piacentini, N. Cosentino, G. Marenzi, P. Poggio, and G.I. Colombo interpreted the data. L. Piacentini and P. Poggio drafted the present work. V.A. Myasoedova, M. Chiesa, C. Vavassori, D. Moschetta, V. Valerio, G. Giovanetti, I. Massaiu, N. Cosentino, G. Marenzi, and G.I. Colombo substantially revised the present study. All authors read and approved the final article.

### Sources of Funding

This work was supported by the Fondazione Umberto Veronesi (Research Grant 2011-12 to G.I. Colombo), the Italian Ministry of Health (Ricerca Corrente Projects Nos 2600696, 2607483, and 2613060, to G.I. Colombo, and Ricerca Finalizzata Projects Nos GR-2018-12366423 to P. Poggio and GR-2019-12370560 to V.A. Myasoedova), and Fondazione Gigi e Pupa Ferrari ONLUS (FPF-14 to P. Poggio).

### Disclosures

None.

### Supplemental Material

Expanded Methods

Major Resources Table

Tables S1–S4

Figures S1–S7

STROBE checklist

Data set

## Supplementary Material
