# Supplementary material for: Whole-Blood Transcriptome Unveils Altered Immune Response in Acute Myocardial Infarction Patients With Aortic Valve Sclerosis
Source: Arterioscler Thromb Vasc Biol. 2023 Dec 21;44(2):452–64. doi: 10.1161/ATVBAHA.123.320106 (PMC10805353; doi:10.1161/ATVBAHA.123.320106)
Supplement: Supplementary file 1 [file atv-44-452-s001.pdf]

**SUPPLEMENTAL MATERIAL**

# Whole-blood transcriptome unveils altered immune response in acute myocardial infarction patients with aortic valve sclerosis

Luca Piacentini[1], Veronika A. Myasoedova[1], Mattia Chiesa[1,2], Chiara Vavassori[1], Donato Moschetta[1], Vincenza Valerio[1], Gloria Giovanetti[1], Ilaria Massaiu[1], Nicola Cosentino[1], Giancarlo Marenzi[1], Paolo Poggio[1,#,¶], Gualtiero I. Colombo[1,#]

1. *Centro Cardiologico Monzino, IRCCS, Milan Italy.*
2. *Department of Electronics, Information and Biomedical Engineering, Politecnico di Milano, Milan, Italy.*

## METHODS

### RNA precipitation and quantification

Five µg of isolated and DNAse-treated RNA were precipitated by ammonium acetate/ethanol method. RNA concentration and quality were assessed, respectively, by micro-volume spectrophotometry on an Infinite M200 PRO Multimode microplate reader (Tecan) and by microfluidics electrophoresis using the RNA 6000 Nano Assay Kit on the 2100 Bioanalyzer system (Agilent Technologies).

### Library preparation and RNA-sequencing

Precipitated total RNA was first depleted for α- and β-globin mRNAs through the GLOBINclear Whole Blood Globin Reduction kit (Thermo Fisher Scientific, Cat. No.: AM1980) and then enriched for the poly(A)+ RNAs using the MicroPoly(A) Purist kit (Thermo Fisher Scientific, Cat. No.: AM1919). Multiplex library RNA Barcoding reagents and Total RNA-Seq kits for the Sequencing by Oligonucleotide Ligation and Detection (SOLiD) System (Applied Biosystems) were used to prepare and pool libraries. Complementary DNA (cDNA) amplification reaction was performed with a 20 µL template in 100-µl final reaction volume for 16 PCR cycles. Library templates clonal amplification was performed on SOLiD p1 DNA beads by emulsion PCR (ePCR) using a 0.5 pM library template and E120 EZbeads scale (Thermo Fisher Scientific, Cat. No.: 4465555). Three different sample libraries were seeded in each lane of a SOLiD flow chip (400 million p2-EZBeads) and templates were paired-end sequenced [75 (forward)-35 (reverse) base pairs (bp)] on a SOLiD 5500xl System (Applied Biosystems).

### Data Processing

XSQTools (Thermo Fisher Scientific) with default parameters was used to remove reads with low quality base values and to generate *.csfasta and *.QV.qual files for each sample. Finally, *.csfasta and *.QV.qual files were converted into *.fastq format. Sequential aligning of raw reads was performed against the GRCh38 Human Genome reference (release 99) with the "*Spliced Transcripts*

*Alignment to a Reference*" (STAR) v2.7.5c software[12], and with Bowtie2 v2.4.1[13] to align locally any reads that were not mapped by STAR. 'Haplotypes' and 'patches' sequences were excluded from the reference, in order to focus on primary assembly and to avoid under-estimation of gene expression. The reference annotation-based transcript (RABT) procedure was implemented, using StringTie suite v2.1.4 to create a new assembly for downstream analysis, integrating the information about known genes and transcripts position in the genome (Ensemble GTF release 99) with those reads mapped in intergenic or intronic regions. Gene expression quantification were computed by featureCounts v2.0.1[14], grouping meta-features by 'gene name'.

**Data adjustment**

Raw count data were first filtered to retain genes with a minimum of 10 counts in at least 20% of the samples. The RUVg method of the RUVSeq R/Bioconductor package[19] was implemented to estimate the factors of "unwanted variation" (k) by including a set of "empirical control genes" identified as the least significantly differentially expressed (DE) genes based on a first-pass DEA performed prior to RUVg. The "empirical control genes" were determined by fitting a statistical model via DESeq2 R/Bioconductor package[18] that included both AMI and AVSc factors (full model) against a null model with only the intercept (*ie* a likelihood ratio test). By this approach, we could exclude genes that were DE for both AMI and AVSc effect (*ie* the pivotal phenotypes in our dataset) thus preventing the recovery of control genes that might instead have exhibited significant differences when comparing different phenotypes in one (or both) of the AMI and AVSc patient groups. After that, the number of k factors was chosen by comparing unadjusted *vs.* adjusted expression data for different numbers of k relying on the use of diagnostic plots, *ie,* relative log expression (RLE) plot, scatter plot of the first two principal components (PC) derived from principal component analysis (PCA) performed on total data, correlation plot between the estimated latent variables and known clinical/technical variables, and histogram of the P-value distribution for testing the differential expression between AVSc *vs.* no-AVSc patients. A k = 13 factors of unwanted variation was chosen in our setting as it showed the best

trade-off between data adjustment and the risk of data overcorrection. Different models for DEA were thus designed and tested to find specific gene expression differences between AVSc *vs.* no-AVSc patients as described in the main manuscript (*cf.* Methods section).

**Feature selection and accuracy**

Feature selection was performed through the *GARS_GA* function of the GARS R/Bioconductor package[17]. Default parameters were used except for: *generat = 500* and *n.gen.conv = 150*; *chr.len* was instead set for a varying length of the feature set, ranging from min=10 to max=200 genes. The input expression matrix for the *GARS_GA* function was obtained from the read counts adjusted for the 13 latent variables identified as above and (log)-transformed though the *DaMiR.normalization* function (DaMiRseq R/Bioconductor package[16], selecting the variance stabilizing transformation (vst) method. The whole, adjusted and log-transformed expression matrix was then subset for the feature set which showed the maximum fitness score and was used as input for the *DaMiR.EnsembleLearning* function for assessing patients' discrimination performance. For this function, the parameters set, other than the default ones, were *iter* = 500 and *cl_type* = c("RF", "SVM", "PLS"). Accuracy was estimated for each classifier by averaging the accuracy obtained from each iteration and weighted through the Ensemble learning method implemented in the DaMiRseq R/Bioconductor package[16].

**Dimensional reduction**

For clustering purpose, the multidimensional scaling (MDS), which helps to visualize the similarity of individual observations of a dataset in a two-dimensional space, was performed through the *cmdscale* function (stats R package) on a distance matrix computed by using the Spearman's correlation dissimilarity expression matrix.

**Functional enrichment analysis by Gene Set Enrichment Analysis (GSEA)**

For GSEA, we used the "*Human_GO_bp_no_GO_iea_symbol.gmt*" gene set collection (release February 2021) from the repository of the Bader Lab (http://download.baderlab.org/EM_Genesets). The GSEA pre-ranked tool option was set. A combined gene rank-score (*cs*), calculated as the product of the log2-FC x -log10(P-value) obtained by the DEA, was used as the gene-ranking metric. The *cs* was employed to weigh the relevance of each gene as a function of both the magnitude (*i.e.*, log2-FC) and the statistical consistency of gene expression differences (*ie* P-value). Other parameters used for analysis included: enrichment statistic=*classic*, number of permutations=*15000* and gene-sets size limit ranging from *min=15* to *max=250*.

## MAJOR RESOURCES TABLE

### Data & Code Availability

| Description | Repository | Persistent ID / URL |
|---|---|---|
| Anonymized RNA-seq | GEO NCBI | https://www.ncbi.nlm.nih.gov/geo/query/acc.cgi?acc=GSE218474 |
| Anonymized metadata and raw data | Zenodo | https://doi.org/10.5281/zenodo.10201186 |
| R codes used for the analyses | GitHub | https://github.com/BioinfoMonzino/Piacentini.et.al.ATVB |

## SUPPLEMENTAL TABLES

### Table S1. Recorded missing data for patient characteristics.

| Variable | Nr. of missing data |
|---|---|
| **Total cholesterol** | 3 |
| **LDL-c** | 4 |
| **HDL-c** | 3 |
| **Triglycerides** | 3 |
| **Glycaemia** | 1 |
| **HbA1c** | 1 |
| **hs-CRP** | 9 |
| **LVEF** | 5 |

LDL-c: low-density lipoprotein cholesterol; HDL-c: high-density lipoprotein cholesterol; HbA1c: hemoglobin A1c; hs-CRP: high-sensitivity C-reactive protein; LVEF: left ventricular ejection fraction

**Table S2. Composite cardiovascular events in patients with and without AVSc.**

| Type of events | AVSc (n=35) | No-AVSc (n=54) |
|---|---|---|
| **Recurrent AMI n (%)** | 2 (5.7) | 5 (9.3) |
| **Recurrent AHF n (%)** | 4 (11.4) | 3 (5.6) |
| **Re-vascularization n (%)** | 8 (14.8) | 11 (20.4) |
| **Cerebrovascular events n (%)** | 3 (8.6) | 1 (1.9) |
| **Cardiovascular mortality n (%)** | 15 (46.9) | 4 (7.4) |
| **Composite outcomes n (%)** | 32 (91.4) | 24 (44.5) |

AMI: acute myocardial infarction; AHF: acute heart failure; AVSc: aortic valve sclerosis

**Table S3. Results of the Cox regression analysis for the full model Mod2.**

| Variable | HR[1] | 95% CI[1] | p-value |
|---|---|---|---|
| **AVSc** | 2.4 | 1.3, 4.5 | 0.007 |
| **AMI type** | 0.7 | 0.4, 1.3 | 0.3 |
| **Age** | 1.0 | 1.0, 1.1 | 0.016 |
| **Previous AMI/PCI/CABG** | 1.2 | 0.6, 2.3 | 0.6 |

[1]HR = Hazard Ratio, CI = Confidence Interval; AMI: acute myocardial infarction; AVSc: aortic valve sclerosis; PCI: Percutaneous Coronary Intervention; CABG: coronary artery bypass grafting

**Table S4. Test of proportional hazard assumption.**

| Variable | Chisq | Df | p-value |
|---|---|---|---|
| **AVSc** | 0.03 | 1 | 0.087 |
| **AMI type** | 2.24 | 1 | 0.13 |
| **Age** | 0.52 | 1 | 0.47 |
| **Previous AMI/PCI/CABG** | 0.67 | 1 | 0.41 |
| **GLOBAL** | 2.53 | 4 | 0.64 |

Hazard assumption for a Cox regression model fit were tested with the Schoenfeld residuals. Test is not statistically significant for each of the variable, and the global test is also not statistically significant.

AMI: acute myocardial infarction; AVSc: aortic valve sclerosis; PCI: Percutaneous Coronary Intervention; CABG: coronary artery bypass grafting.

## SUPPLEMENTAL FIGURES

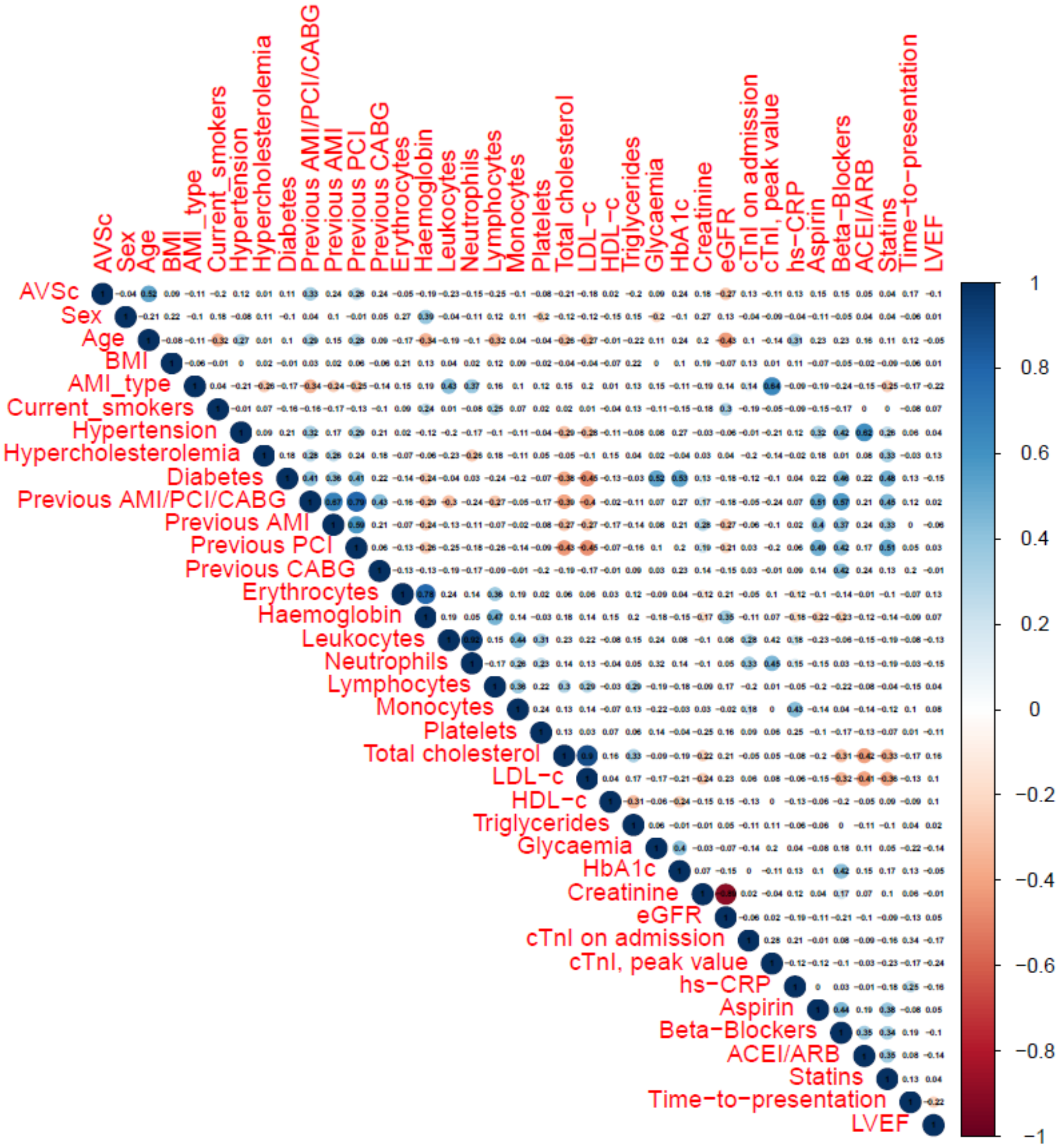


**Figure S1. Correlation plot.** Spearman's correlation plot shows correlations among clinical variables. Positive and negative significant correlations (P-Value<0.01) are marked with colored circle (blue and red, respectively). Numbers refer to correlation coefficient and color gradient is proportional to correlation values. AVSc, aortic valve sclerosis; BMI, body mass index; STEMI, ST-segment elevation myocardial infarction; AMI, acute myocardial infarction; PCI, percutaneous coronary intervention; CABG, coronary artery bypass grafting; LDL-c, low-density lipoprotein cholesterol; HDL-c, high-density lipoprotein cholesterol; HbA1c, hemoglobin A1c; eGFR, estimated glomerular filtration rate, based on the Modification of Diet in Renal Disease equation; cTnI, cardiac troponin I; hs-CRP, high-sensitivity C-reactive protein; ACEI, angiotensin-converting enzyme inhibitor; ARB, angiotensin-II receptor blocker; LVEF, left ventricular ejection fraction.

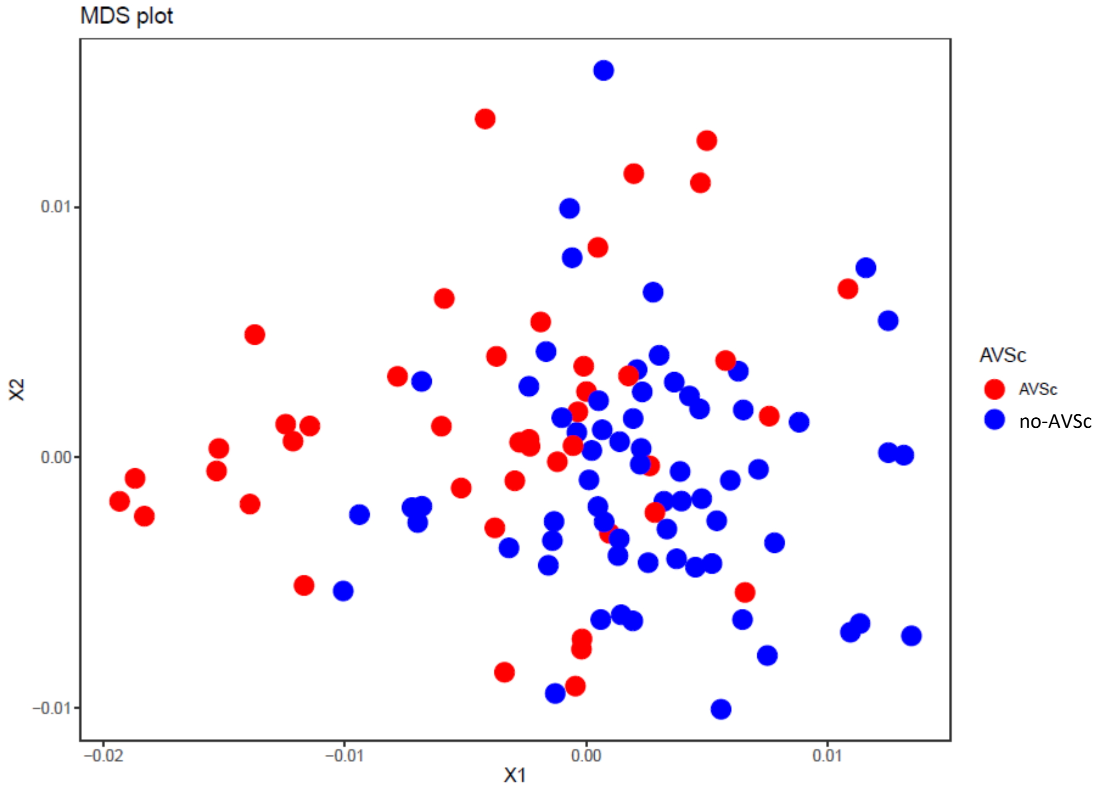


**Figure S2. MDS plot.** Scatterplot of the two coordinates (X1 and X2) obtained from the multidimensional scaling performed on the whole gene-expression dataset. Colors refer to AVSc and no-AVSc patients (red and blue, respectively).

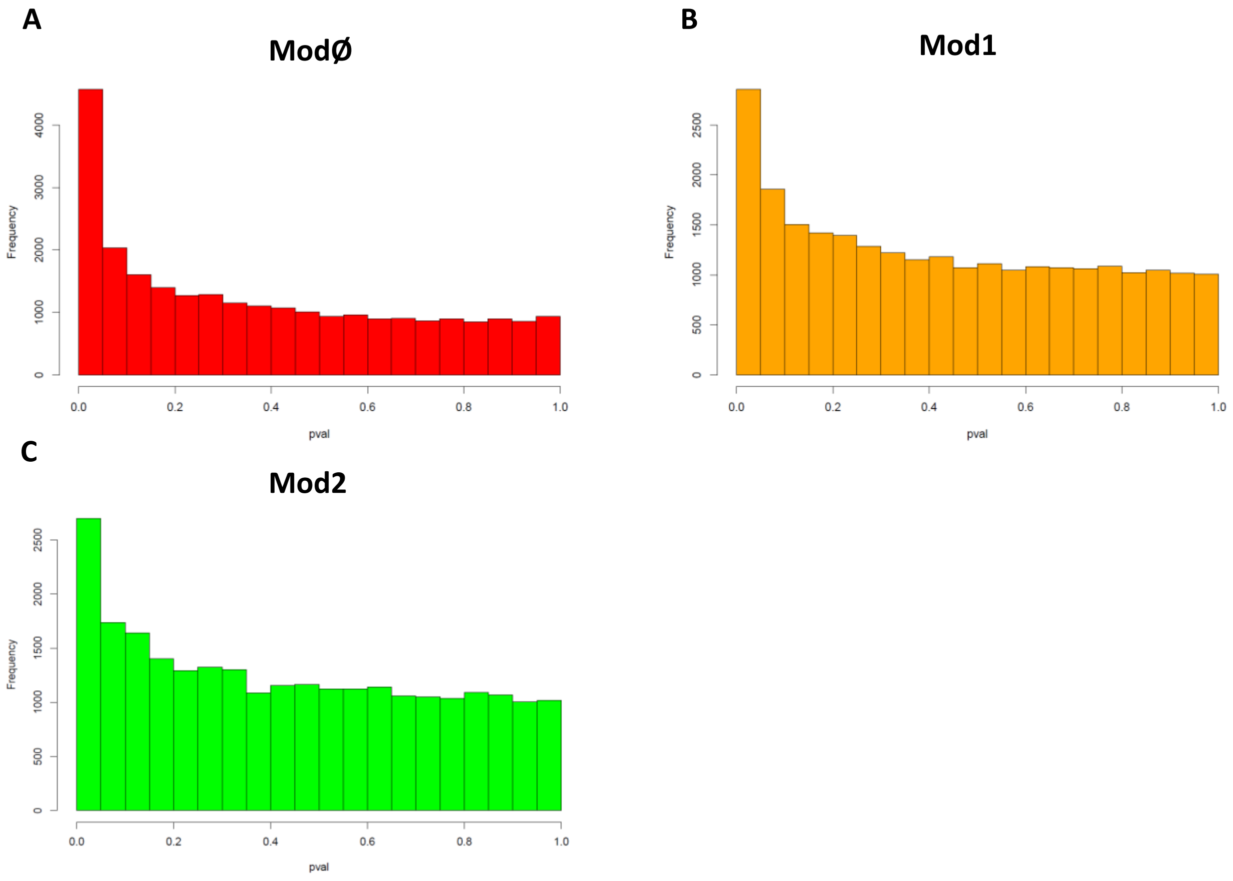


**Figure S3. Histogram of P-Value distribution.** The histogram of the distribution of P-Values for non-DE genes would be ideally uniformly distributed across the unit interval, whereas the P-Values for DE genes present a spike near zero. The comparison between AVSc *vs.* no-AVSc applying ModØ (**A**), Mod1 (**B**), Mod2 (**C**) shows the expected shape of the histogram of the P-Values for truly DE genes.

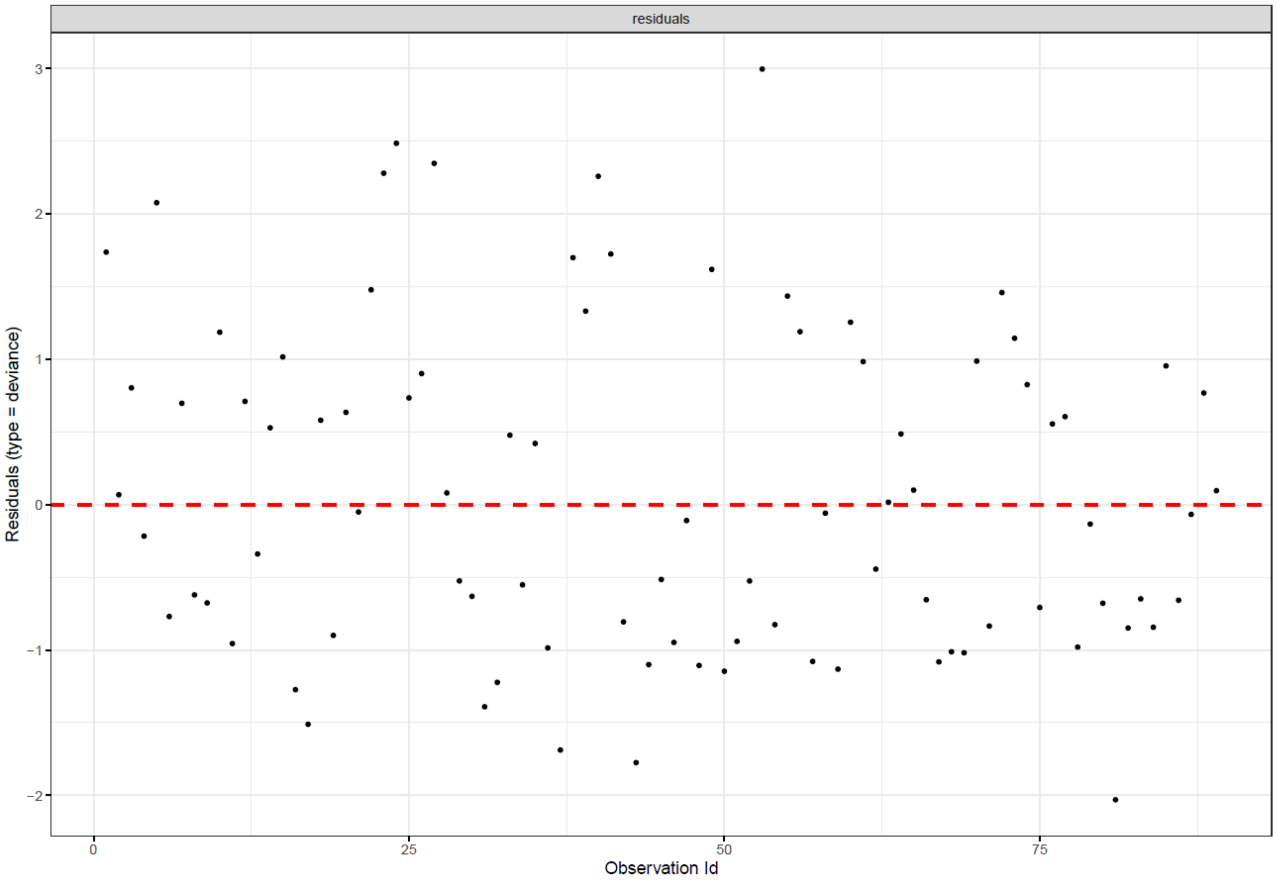


**Figure S4. Testing outliers.** Deviance residuals, which represent a normalized transform of the martingale residual, are plotted to detect possible outliers. The index plot was generated using the ggcoxdiagnostics function of the survminer R package, setting the type="deviance" argument. The graph shows that the residuals were symmetrically distributed around zero and did not substantially present very large or small values.

A

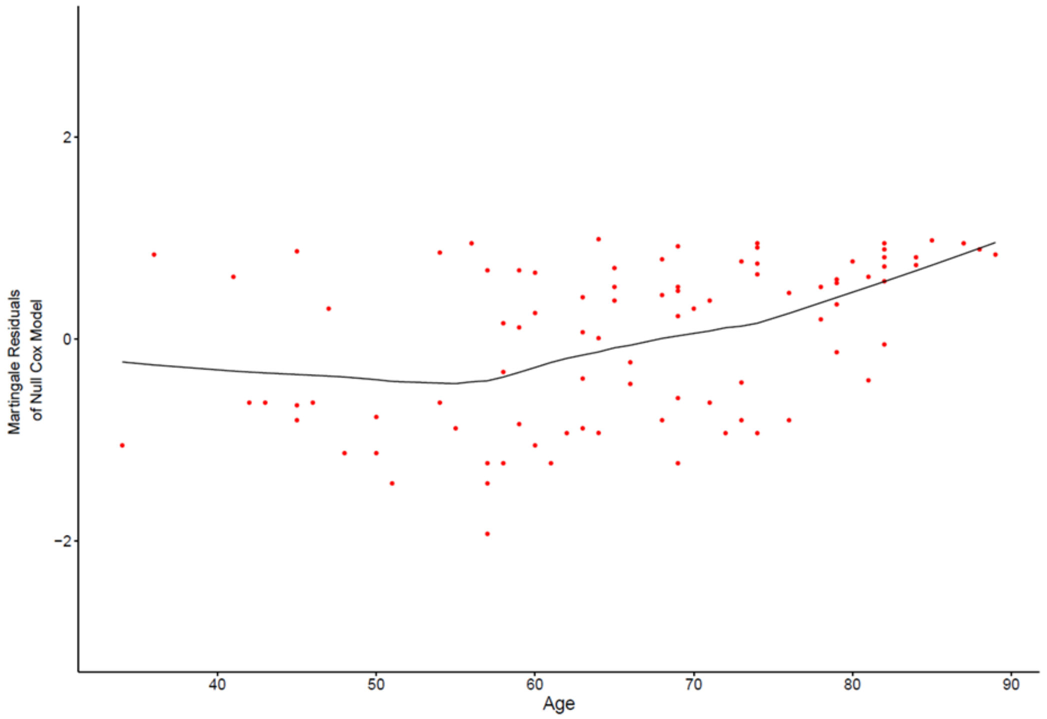


B

| Variable | HR[1] | 95% CI[1] | p-value |
|---|---|---|---|
| AVSc | 2.5 | 1.3, 4.8 | 0.005 |
| AMI type | 0.7 | 0.41, 1.36 | 0.3 |
| ns(Age, df = 2)1 | 0.8 | 0.05, 13.7 | 0.9 |
| ns(Age, df = 2)2 | 7.3 | 2.2, 24.9 | 0.001 |
| Previous AMI/PCI/CABG | 1.2 | 0.6, 2.2 | 0.6 |

**Figure S5. Testing non linearity.** The Cox proportional model included "Age" as the only continuous variable. (**A**) The plot shows the Martingale residuals of null cox proportional hazards model against "Age" to detect possible non-linearity, drawn using the ggcoxfunctional function of the survminer R package. The fitted line with lowess function shows a slight non-linear trend, although we do not believe that the global model is significantly affected by non-linear bias. (**B**) Indeed, when we included a spline term for "Age" in the model, we showed that AVSc still had an increased risk of cardiovascular events, even after all these adjustments (see also **Table S3** for model comparison).

[1]HR = Hazard Ratio, CI = Confidence Interval; AMI: acute myocardial infarction; AVSc: aortic valve sclerosis; PCI: Percutaneous Coronary Intervention; CABG: coronary artery bypass grafting; ns= natural spline; df=degrees of freedom.

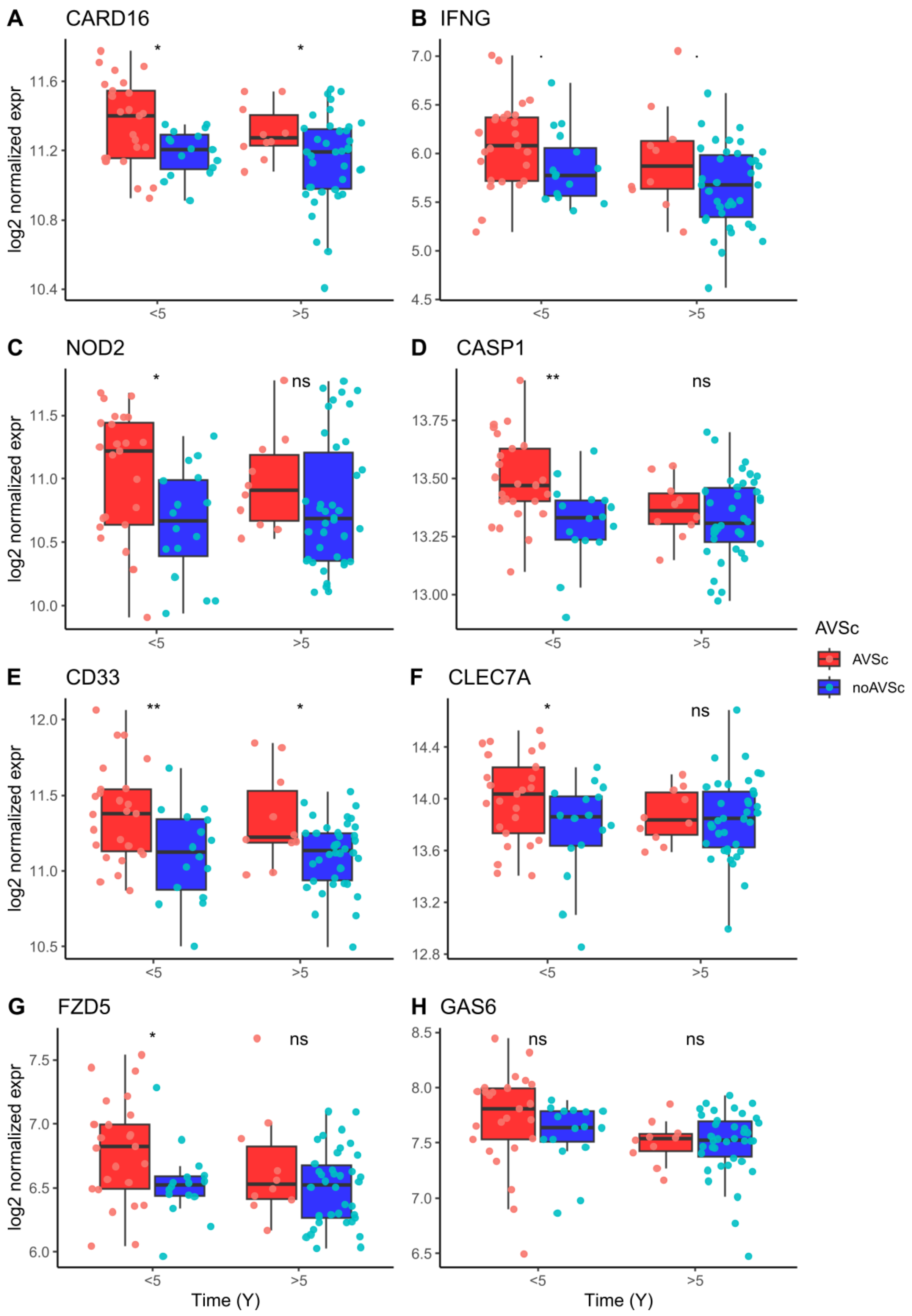


**Figure S6. Box plots of relevant positive regulation of interleukin-1 beta.** Box plots show the differences over the time-to-cardiovascular event (<5 and >5 years; x-axis) of the normalized expression values (y-axis) of the positive

regulation of interleukin-1 beta genes in AVSc and no-AVSc patients assessed on blood sample collected at hospital presentation. The eight genes shown (A to H) were selected as they presented the highest combined-ranked score (cs; i.e., log2-FC x -log10[P-value]) among the platelet activation/aggregation genes as resulted by the differential expression analysis of AVSc vs. no-AVSC in the full adjusted statistical model (Mod2). Number of patients for time-to-cardiovascular event comparisons were for <5 years: AVSc=25, no-AVSc=16; and for >5 years, AVSc=10, no-AVSc=38. Box (red and blue) and dots (pink and light blue) colors refer to AVSc and no-AVSc patients, respectively. Stars mark significant differences for post-hoc tests with P-Values: **<0.01; *<0.05; •<0.1; ns=non-significant difference.

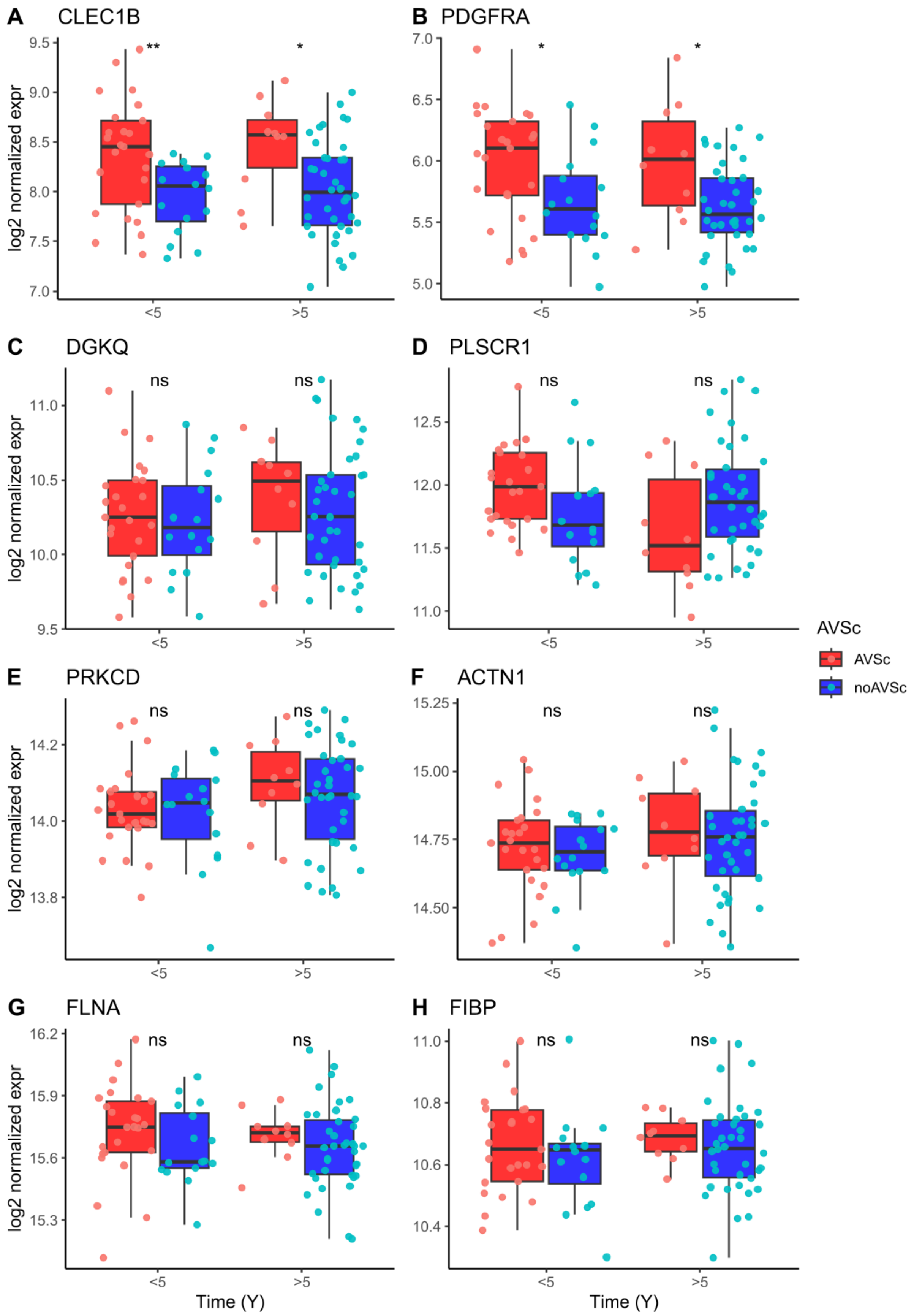


**Figure S7. Box plots of relevant platelet activation/aggregation genes.** Box plots show the differences over the time-to-cardiovascular event (<5 and >5 years; x-axis) of the normalized expression values (y-axis) of the platelet

activation/aggregation genes in AVSc and no-AVSc patients assessed on blood sample collected at hospital presentation. The eight genes shown (A to H) were selected as they presented the highest combined-ranked score (cs; i.e., log2-FC x -log10[P-value]) among the platelet activation/aggregation genes as resulted by the differential expression analysis of AVSc vs. no-AVSC in the full adjusted statistical model (Mod2). Number of patients for time-to-cardiovascular event comparisons were for <5 years: AVSc=25, no-AVSc=16; and for >5 years, AVSc=10, no-AVSc=38. Box (red and blue) and dots (pink and light blue) colors refer to AVSc and no-AVSc patients, respectively. Stars mark significant differences for post-hoc tests with P-Values: **<0.01; *<0.05; •<0.1; ns=non-significant difference.

**STROBE checklist**

| | Item No | Recommendation | Page No |
|---|---|---|---|
| **Title and abstract** | 1 | (*a*) Indicate the study's design with a commonly used term in the title or the abstract | 1 |
| | | (*b*) Provide in the abstract an informative and balanced summary of what was done and what was found | 1 |
| **Introduction** | | | |
| Background/rationale | 2 | Explain the scientific background and rationale for the investigation being reported | 2 |
| Objectives | 3 | State specific objectives, including any prespecified hypotheses | 2-3 |
| **Methods** | | | |
| Study design | 4 | Present key elements of study design early in the paper | 3 |
| Setting | 5 | Describe the setting, locations, and relevant dates, including periods of recruitment, exposure, follow-up, and data collection | 3 |
| Participants | 6 | (*a*) *Cohort study*—Give the eligibility criteria, and the sources and methods of selection of participants. Describe methods of follow-up<br>*Case-control study*—Give the eligibility criteria, and the sources and methods of case ascertainment and control selection. Give the rationale for the choice of cases and controls<br>***Cross-sectional study*—Give the eligibility criteria, and the sources and methods of selection of participants** | 3 |
| | | (*b*) *Cohort study*—For matched studies, give matching criteria and number of exposed and unexposed<br>*Case-control study*—For matched studies, give matching criteria and the number of controls per case | |
| Variables | 7 | Clearly define all outcomes, exposures, predictors, potential confounders, and effect modifiers. Give diagnostic criteria, if applicable | 3-4 |
| Data sources/ measurement | 8 | For each variable of interest, give sources of data and details of methods of assessment (measurement). Describe comparability of assessment methods if there is more than one group | 4-6 |
| Bias | 9 | Describe any efforts to address potential sources of bias | 6 |
| Study size | 10 | Explain how the study size was arrived at | 7 |
| Quantitative variables | 11 | Explain how quantitative variables were handled in the analyses. If applicable, describe which groupings were chosen and why | 7-8 |
| Statistical methods | 12 | (*a*) Describe all statistical methods, including those used to control for confounding | 6-7 |
| | | (*b*) Describe any methods used to examine subgroups and interactions | N/A |
| | | (*c*) Explain how missing data were addressed | 7 |
| | | (*d*) *Cohort study*—If applicable, explain how loss to follow-up was addressed | N/A |

| | | | |
|---|---|---|---|
| | | *Case-control study*—If applicable, explain how matching of cases and controls was addressed<br>***Cross-sectional study*—If applicable, describe analytical methods taking account of sampling strategy** | |
| | | (*e*) Describe any sensitivity analyses | N/A |
| **Results** | | | |
| Participants | 13 | (a) Report numbers of individuals at each stage of study—eg numbers potentially eligible, examined for eligibility, confirmed eligible, included in the study, completing follow-up, and analysed | 9 |
| | | (b) Give reasons for non-participation at each stage | 12 |
| | | (c) Consider use of a flow diagram | N/A |
| Descriptive data | 14 | (a) Give characteristics of study participants (eg demographic, clinical, social) and information on exposures and potential confounders | 8 |
| | | (b) Indicate number of participants with missing data for each variable of interest | 25 |
| | | (c) *Cohort study*—Summarise follow-up time (eg, average and total amount) | N/A |
| Outcome data | 15 | *Cohort study*—Report numbers of outcome events or summary measures over time | N/A |
| | | *Case-control study*—Report numbers in each exposure category, or summary measures of exposure | N/A |
| | | ***Cross-sectional study*—Report numbers of outcome events or summary measures** | 12 |
| Main results | 16 | (*a*) Give unadjusted estimates and, if applicable, confounder-adjusted estimates and their precision (eg, 95% confidence interval). Make clear which confounders were adjusted for and why they were included | 12 |
| | | (*b*) Report category boundaries when continuous variables were categorized | N/A |
| | | (*c*) If relevant, consider translating estimates of relative risk into absolute risk for a meaningful time period | N/A |
| Other analyses | 17 | Report other analyses done—eg analyses of subgroups and interactions, and sensitivity analyses | 13 |
| **Discussion** | | | |
| Key results | 18 | Summarise key results with reference to study objectives | 13 |
| Limitations | 19 | Discuss limitations of the study, taking into account sources of potential bias or imprecision. Discuss both direction and magnitude of any potential bias | 16-17 |
| Interpretation | 20 | Give a cautious overall interpretation of results considering objectives, limitations, multiplicity of analyses, results from similar studies, and other relevant evidence | 17 |
| Generalisability | 21 | Discuss the generalisability (external validity) of the study results | 16-17 |
| **Other information** | | | |
| Funding | 22 | Give the source of funding and the role of the funders for the present study and, if applicable, for the original study on which the present article is based | 18 |
